# Determinants of Renal Micro-Perfusion as Assessed with Contrast-Enhanced Ultrasound in Healthy Males and Females

**DOI:** 10.3390/jcm12124141

**Published:** 2023-06-20

**Authors:** Antonio Ulpiano Trillig, Aikaterini Damianaki, Mariëlle Hendriks-Balk, Wendy Brito, Jonas Garessus, Michel Burnier, Grégoire Wuerzner, Menno Pruijm

**Affiliations:** Service of Nephrology and Hypertension, Lausanne University Hospital and University of Lausanne, Rue du Bugnon 17, 1005 Lausanne, Switzerland; antonio.ulpiano@gmail.com (A.U.T.); aikaterini.damianaki@chuv.ch (A.D.); marielle.hendriks-balk@chuv.ch (M.H.-B.); wendy.brito@chuv.ch (W.B.); jonas.garessus@chuv.ch (J.G.); michel.burnier@chuv.ch (M.B.); gregoire.wuerzner@chuv.ch (G.W.)

**Keywords:** contrast-enhanced ultrasound, renal microcirculation, perfusion index, kidney disease, imaging technologies, plasma renin activity, sex

## Abstract

(1) Background: The renal microcirculation is essential to maintain the renal function, but its determinants in humans have been poorly studied. Contrast-enhanced ultrasound (CEUS) allows the non-invasive quantification of the cortical micro-perfusion at the bedside using the perfusion index (PI). The aims of this study were to assess whether differences exist in PI between healthy males and females and to identify clinical determinants associated with cortical micro-perfusion. (2) Methods: Healthy, normotensive volunteers (eGFR > 60 mL/min/1.73 m^2^, no albuminuria) underwent CEUS under standardized conditions with the destruction–reperfusion (DR) technique. The mean PI of four DR sequences was reported as the primary outcome measure (3) Results: A total of 115 subjects (77 females and 38 males) completed the study; the mean ± SD age was, respectively, 37.1 ± 12.2 and 37.1 ± 12.7 years in females and males, and the mean eGFR was 105.9 ± 15.1 and 91.0 ± 17.4 mL/min/1.73 m^2^. The PI (median) was higher in females than in males, i.e., 2705 (IQR 1641–3777) vs. 1965 (IQR 1294–3346) arbitrary units (a.u), *p* = 0.02). A correlation analysis showed positive associations between PI and eGFR, female sex, heart rate, plasma renin activity (PRA) and plasma aldosterone concentrations (PAC), negative associations with potassium, bicarbonate and systolic blood pressure, and no associations with age, body mass index and renal resistive index (RRI). In a multivariate linear regression analysis, only PRA remained significantly associated with PI. (4) Conclusions: Although the PI was higher among females, this association was no longer significant after adjustment for covariates. There was no difference in females tested during the follicular or the luteal phases. In conclusion, the PI was only weakly influenced by classic clinical variables, but was positively associated with PRA, suggesting that the renin–angiotensin system plays a role in the regulation of the cortical micro-perfusion in humans. Identifying which other factors contribute to the large variations in micro-perfusion across individuals needs further study.

## 1. Introduction

The kidneys are extremely well perfused organs. They receive ~20% of the cardiac output, although they account for less than 1% of total body weight. Global kidney perfusion (macro-circulation) and its impact on the development of renal dysfunction have been well studied in both acute and chronic settings, thanks to the availability of many techniques such as para-amino-hippurate (PAH) clearance, renal Doppler ultrasound (US), computed tomography (CT) and Magnetic Resonance Imaging (MRI) [1,2,3]. The kidneys also contain an extensive micro-circulatory network that includes glomerular and peri-tubular capillaries. Animal studies with micro-CT and, more recently, micro-fill techniques [4] have demonstrated that dysfunction and rarefaction of the micro-vessels play a prominent role in the induction of renal injury. In addition, it has been stated that a defective renal microcirculation is a universal pathological feature of patients with chronic kidney disease (CKD) [5,6]. Arterial hypertension (AHT) and diabetes mellitus are major causes of CKD and are indeed characterized by endothelial dysfunction and microvascular damage [5].

Therefore, a deeper understanding of the physiological regulation of renal micro-perfusion and the impact of its dysregulation on the development and progression of kidney diseases is crucial. However, this has been hampered by the lack of non-nephrotoxic and non-invasive techniques to quantify and visualize the renal microcirculation in humans, and data mainly come from biopsy studies.

Interestingly, several epidemiological studies showed that females progress less rapidly to end-stage renal disease [7,8]. This may be due to lifestyle differences, but many believe that hormones also play an important role, possibly due to interferences with renal hemodynamics. Some studies found that the renal plasma flow and the glomerular filtration rate (GFR) are higher during the luteal phase than during the follicular phase [9,10]. However, whether differences exist between males and females in the renal microcirculation and between females according to the phase of the menstrual cycle has, to the best of our knowledge, not been studied in detail.

Contrast-enhanced ultrasound (CEUS) has been classically used to characterize cysts and other renal structural lesions, but we and others have shown that it can also be used to quantify the cortical microcirculation. With CEUS, non-nephrotoxic microbubbles with the size of human red blood cells are injected in the circulation. The microbubbles remain intra-vascular and are not filtered but are eliminated within thirty minutes by the liver and lungs. Intermittent application of high-intensity sound waves to a kidney will destroy the microbubbles locally; their reappearance rate in the cortex can be measured and expressed as the perfusion index PI. As such, the PI is a quantitative measure of cortical micro-perfusion [11]. By applying this destruction–replenishment (DR) technique, we recently showed that patients with CKD have a much lower cortical micro-perfusion than persons without CKD [12]. Similar results were found by Han et al. with a slightly different CEUS technique [13]. Despite these findings, little remains known about the clinical determinants of the PI in health and disease.

Therefore, the goal of our study was to provide insight in the clinical and biological determinants of the renal microcirculation as assessed by CEUS in healthy volunteers, with a particular focus on sex differences. We also studied whether females at different phases of their menstrual cycle exhibit differences in renal micro-perfusion.

## 2. Materials and Methods

### 2.1. Study Population

This study included all the healthy volunteers that participated in the GenderBOLD (NCT04085094) or in the BRAIrdN study (NCT03473275). These monocentric studies took both place in the Service of Nephrology and Hypertension of the Lausanne University Hospital between 2017 and 2022. The primary aim of the GenderBOLD study was to assess the influence of the menstrual cycle and menopause on renal tissue oxygenation, renal hemodynamics and sodium handling under different dietary sodium conditions using, respectively, BOLD-MRI, CEUS and ^23^Na MRI in healthy controls and patients with CKD. GenderBOLD included a larger proportion of females than males. The BRAIrdN study used CEUS to assess the renal hemodynamic response to a cold pressor test in healthy controls and in patients with arterial hypertension (AHT).

For this study, we analyzed the CEUS data acquired from healthy controls of the GenderBOLD and BRAIrdN studies. None of the healthy volunteers participated to both studies.

Details of the two studies have been described previously [12,14]. In brief, the inclusion criteria for all the healthy controls in both studies were: age ≥ 18 years, being normotensive (office blood pressure < 135/85 mmHg) and not taking any medications. The exclusion criteria for the healthy volunteers were the presence of CKD (defined as eGFR < 60 mL/min/1.73 m^2^ and/or albuminuria > 300 mg/d) or arterial hypertension, an acute or chronic disease state that potentially influenced the renal function, pregnancy, a known allergy to the ultrasound contrast agent, renal cysts at renal US screening, liver disease or heart failure.

All the participants signed an informed consent. Both studies were approved by the ethical committee of the Canton de Vaud and conducted following the principles of the declaration of Helsinki.

### 2.2. Study Design and Settings

This was an observational study that analyzed the combined data from healthy volunteers participating in the GenderBOLD or the BRAIrdN studies and who underwent a CEUS exam.

In the GenderBOLD cohort, the volunteers underwent renal CEUS during two visits, firstly under a high-salt (HS) diet and secondly under a low-salt (LS) diet. For pre-menopausal females, the two visits were performed each time during the same phase of their menstrual cycle (follicular or luteal).

In the BRAIrdN cohort, the volunteers underwent one CEUS exam before and after a cold pressor test, without particular salt diet recommendations. For the purpose of this analysis, we only included CEUS exams performed before the cold pressor test.

Each study visit took place under standardized conditions, after an overnight fast, in the morning. All the participants were instructed to avoid high-intensity exercise the day before and on the day of the study visits. A venous catheter was placed in an antecubital vein for blood sampling and injection of the Sonovue^®^ contrast product. The CEUS exams were performed after thirty minutes of rest in the supine position.

In the GenderBOLD cohort, blood pressure (BP) was measured in the supine position after 15 min of rest, at the brachial level using a validated device (Omron HEM-907) according to the international guidelines [15]. In the BRAIrdN cohort, BP was also measured in the supine position after 15 min of rest using the brachial cuff of the Finapres^®^ NOVA device (Finapres Medical Systems, Enschede, The Netherlands). Body weight was measured using a validated Seca^®^ scale.

### 2.3. Laboratory

Blood samples were drawn for the measurement of electrolytes, urea, creatinine and uric acid. The glomerular filtration rate was estimated by the CKD-EPI formula (eGFR). Plasma renin activity (PRA) and plasma aldosterone concentration (PAC) were measured in the supine position after 30 min; the samples were collected on the venous catheter placed previously. PRA was determined by a radioimmunoassay kit for the quantitative determination of Angiotensin I in human plasma (REN-CT2, Cisbio Bioassays, Codolet, France), and PAC was determined using a commercial RIA kit (Aldo-Riact; CIS Bio International, Yvette, Cedex, Saclay, France).

For the GenderBOLD study, a 24 h urine collection for Na^+^ was performed the day prior to the visit, and a dipstick urinalysis on the day of the visit. The pre-menopausal females had an additional measurement of the estradiol and progesterone concentrations.

Finally, in the GenderBOLD cohort, the mean of the different variables was calculated based on the two visits, under HS and LS conditions. For the volunteers of the BRAIrdN cohort, the means were calculated based on a unique visit.

### 2.4. Renal Ultrasound Parameters

The volunteers underwent a renal doppler US followed by CEUS. All exams were performed by two experienced sonographers (M.P. and W.B.), using a Samsung RS80A device. The kidney volume was measured using the ellipsoid formula. The Doppler mode was applied in order to select the segmental artery with the easiest accessibility and highest quality of Doppler waveforms for the measurement of the renal resistive index (RRI). For this reason, in almost all participants, the right kidney was chosen. The measurements of RRI were performed in expiratory breath hold and calculated as (peak systolic velocity—end diastolic velocity)/peak systolic velocity in the color Doppler ultrasound mode.

For the CEUS exam, the probe was oriented in such a way that an optimal long axis view of the right kidney was obtained, in order to assure a large cortical surface area. The Sonovue^®^ contrast agent was injected as a continuous infusion with a special rotating syringe pump (Vueject^®^, Bracco SA, Milano, Italy) at a continuous rate of ~1 mL/min (0.015 mL/kg/min).

Image depth, focus, gain and frame rate were optimized and held constant during the experiment. To quantify the intra-renal perfusion, the destruction–replenishment (DR) technique was used as described previously [11,12].

Briefly, the rate at which the microbubbles replenish the renal tissue after destruction by pulses at high MI is proportional to the local blood flow and is expressed as time–intensity curves (TICs). The division of the intensity of the signal (expressed as regional blood volume, RBV) by the mean transit time (mTT) allows the calculation of the PI as a proxy of the renal microcirculation (PI = rBV/mTT) [12]. Four consecutive DR sequences were performed at each time point in breath hold, in order to avoid movement artefacts.

The PI was calculated as the mean of 4 values of the DR sequences. For the participants of the GenderBOLD cohort, the PI used for the analysis was derived from the mean PI values of the two study visits (HS/LS). In the case of missing data in one of the two visits, we only used the PI value from the other visit, as our initial analysis demonstrated that the PI was not different between HS/LS conditions.

### 2.5. Statistics

The data are expressed as means ± standard deviation (SD) or as median (interquartile range), as appropriate. Comparisons between the baseline characteristics of the study groups (males and females) and between participants from the GenderBOLD and BRAIrdN cohorts were performed using *t*-test and Mann–Whitney Wilcoxon Test, depending on the normality of the distribution. Correlations between the PI and the variables of interest were assessed by the Spearman test or by the point biserial correlation test to test the correlation between PI and sex or menstrual phase, followed by univariate and multivariate regression analysis to account for possible effects of the variables previously shown to be associated with the PI in univariate analysis. Log transformation was used for variables that were not normally distributed (PI, PRA and PAC). A two-sided *p*-value < 0.05 was considered statistically significant. All statistical analyses were performed with Stata 14 (Statacorp, College Station, TX, USA).

The PI was the main outcome variable of the study. By hypothesizing a difference of the PI of 10% between healthy males and females, with a standard deviation of 10%, and considering the higher number of females compared to males, 26 females and 13 males had to be included to have a power of 80%.

## 3. Results

### 3.1. Participants and Baseline Characteristics

From an initial sample of 117 healthy volunteers, a total of 115 volunteers were included, of which 77 were females, and 38 were males. Two participants were excluded as they had a PI superior to 10.000 a.u. (99% CI). Technical errors in the Sonovue^®^ administration and dose were detected in those patients.

A summary of the baseline characteristics is shown in Table 1. Age and BMI were similar between females and males. Females had a higher heart rate (HR) but lower systolic blood pressure (SBP) and potassium and bicarbonate levels than males. Females had higher eGFR, PRA and PAC than males. There were no significant differences in eGFR between the GenderBOLD and the BRAIrdN cohorts (Supplementary Table S1).

### 3.2. Doppler US and CEUS Parameters

The kidney volume was larger in males. The median and mean PI were significantly higher in females, whereas there was no difference in rBV and mTT (see Table 2).

In the GenderBOLD cohort, 14 participants had missing values for the PI in the HS visit, and 18 participants in the LS visit. For these patients, we took the available PI, instead of the mean PI from the two visits.

**Figure 1:**
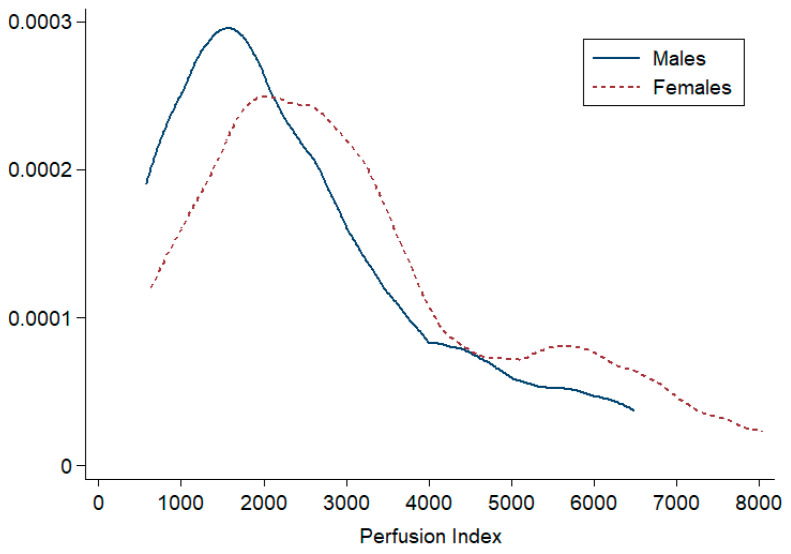
Kernel density plot showing the distribution of the Perfusion Index by sex.

The distribution of the PI was large and skewed in both males and females (Figure 1). The PI values of the GenderBOLD and BRAIrdN cohorts showed similar distributions (see Supplementary Figure S1).

We found no differences in the renal US parameters in pre-menopausal females tested during the follicular or the luteal phase. The results are presented in Table 3.

### 3.3. Associations between CEUS Indices and Clinical and Laboratory Variables

The correlation analyses (Spearman tests) between the PI and the clinical and biological variables of interest are shown in Supplementary Table S2.

**Figure 2:**
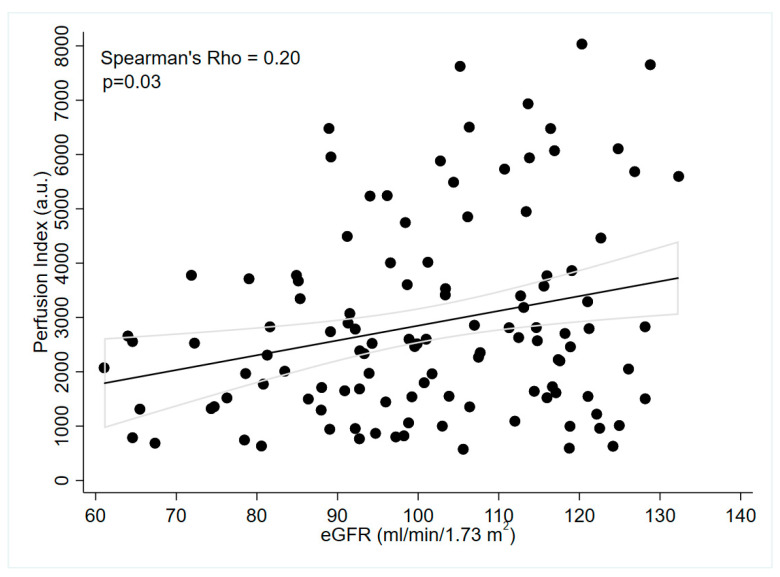
Relationship between Perfusion Index and eGFR in the total population.

In brief, a positive association was found between PI and eGFR (rho = 0.20; *p* = 0.03) (Figure 2). The PI was also correlated with female sex (point biserial coefficient 0.20; *p* = 0.03). There were no significant correlations between the PI and the different phases of the menstrual cycle, nor with the estradiol and progesterone levels in pre-menopausal females. There was no significant difference of PI between pre- and post-menopausal females. We also found no association between PI and BMI or age.

Regarding the systemic and renal US parameters, the PI was positively correlated with HR (rho = 0.25; *p* = 0.01) and inversely correlated with SBP (rho= −0.19; *p* = 0.04). No associations were found between PI and RRI or kidney volume.

**Figure 3:**
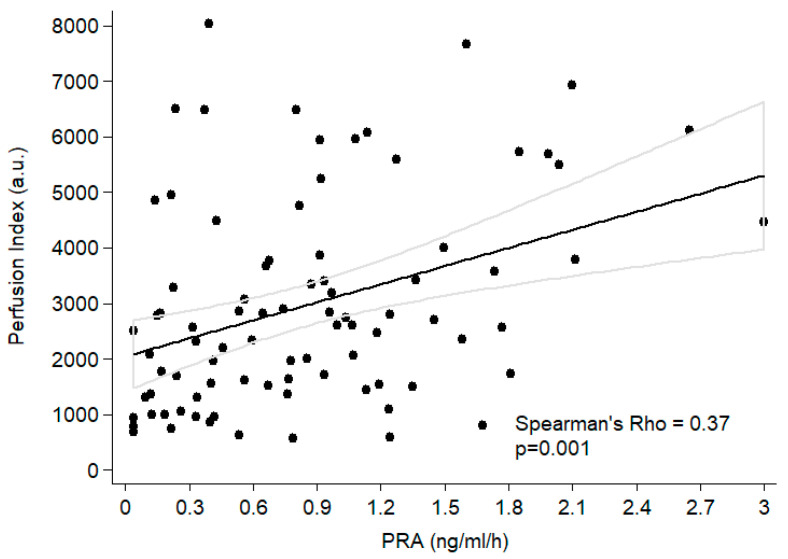
Relationship between Perfusion Index and PRA in the total population.

The PI correlated with PAC (rho = 0.22; *p* = 0.04), PRA (rho = 0.37; *p* = 0.00) (Figure 3) and the potassium (rho= −0.24; *p* = 0.01) and bicarbonate (rho= −0.20; *p* = 0.03) levels.

In a second step, univariate and multivariate regression analyses were performed. The univariate analysis showed the same associations as the Spearman correlation analysis. The multivariate analysis included all the variables that correlated significantly with the PI in univariate analysis, except for PAC which was too strongly correlated with PRA (Rho = 0.58) to be integrated in the model. The association between PI and sex was no longer significant after adjustment for eGFR. The results of the multivariable regression analysis are shown in Table 4. In the fully adjusted model, the PI was only associated with PRA but no longer with the other above-mentioned parameters.

We compared the mean values of PRA in the HS/LS visits of the GenderBOLD and BRAIrdN cohorts. The PRA mean values were not significantly different between the HS visits of the GenderBOLD and those of the BRAIrdN studies (0.53 ± 0.43 ng/mL/h vs. 0.51 ± 0.41 ng/mL/h, *p* = 0.78).

In a separate analysis using only the PI values of the HS visits of GenderBOLD and the values of BRAIrdN for all variables, only eGFR, bicarbonates and PRA (Log) were associated with the PI (Log) in univariate analysis. No association remained significant in the multivariate analysis using the correction with eGFR, bicarbonates and PRA, although there was a tendency towards an association of the PI with eGFR (*p* = 0.068). The results are shown in Supplementary Table S3. Of note, in this analysis the sample size was smaller (101 patients: 68 females and 33 males).

Finally, we conducted also a sub-analysis of the correlation of PAC with the PI. The mean PAC levels were significantly different between the HS/LS visits of GenderBOLD and the BRAIrdN visits. There was a significant positive correlation between PI and PAC for the LS diet of the GenderBOLD cohort (r = 0.27; *p* = 0.013), and a non-significant negative correlation for the HS diet (r= −0.11; *p* = 0.32). In the BRAIrdN study, the correlation was positive but not significant.

## 4. Discussion

Taken together, in this study the CEUS-assessed perfusion index (PI) as a proxy of cortical micro-perfusion was higher in females than in males, at least in unadjusted analyses. The phases of menstrual cycle and the estradiol and progesterone levels did not have an impact on the cortical micro-perfusion. The PI was positively associated with eGFR and PRA and negatively associated with the bicarbonate and potassium levels. The PI was not associated with RRI but was negatively associated with SBP, suggesting that the renal microcirculation is (at least partly) independent of the macro-circulation in healthy males and females.

In multivariate analysis, only PRA remained positively associated with the PI, underlining the importance of the renin–angiotensin system in the regulation of the renal micro-circulation.

Finally, the large variation in the PI between individuals remains largely unexplained by the clinical and biological variables included in our analyses and calls for further studies to establish the unidentified factors involved.

In this study, the CEUS-assessed cortical PI micro-perfusion was higher in females than in males. We are not aware of any previous studies in humans that compared specifically the PI between males and females. In the study by Schneider et al., solely male subjects were included [11]. A study by Dong et al. included nine females subjects with suspected CKD, but no healthy controls [16]. A previous CEUS study performed by our team included only 40 healthy controls (62.5%), which hampered definite conclusions.

Another non-invasive, non-nephrotoxic technique that allows the assessment of the cortical micro-perfusion in humans is Arterial Spin Labelling-Magnetic Resonance Imaging (ASL-MRI). Results of ASL-MRI studies show, like those of CEUS, that renal cortical perfusion is reduced in CKD patients compared with healthy volunteers [17]. Of note, in one of the largest cohorts studied by renal ASL-MRI and with female participation reaching 71% in the healthy control group, the effect of sex on kidney perfusion found in diabetic patients along different stages of CKD compared to healthy controls was not significant [18]. However, this study included only 46 healthy controls, with a mean age of 60 years.

The higher PI in females is interesting, as it may explain why females have, according to BOLD-MRI studies, higher levels of cortical oxygenation [19]. Higher cortical micro-perfusion and oxygenation may also provide some insight in the reasons why females evolve less frequently to end-stage kidney disease, but this remains highly speculative. However, it is important to underline that the PI was no longer associated with female sex in the multivariate analysis. Therefore, the higher PI in females was possibly the result of differences in baseline characteristics. The clinical characteristics age and BMI were well matched in our study, but eGFR was significantly higher in females, and their SBP was lower than in males. Indeed, as soon as we included eGFR in the regression model, the PI was no longer associated with sex. However, one may wonder whether this is an over-adjustment, as the glomerular filtration rate is directly dependent on glomerular perfusion.

The difference in eGFR is important, as eGFR was positively associated with the PI in our study. Similar associations were found in previous studies. Srivastava et al. reported, for example, that the PI was inversely related to the CKD stages until end-stage kidney disease [20]. In the aforementioned study from our group by Garessus et al., a nonlinear association between PI and eGFR was found: the relationship was flat at eGFR values < 60 mL/min/1.73 m^2^ and positive at eGFR values ≥ 60 mL/min/1.73 m^2^, suggesting that a considerable amount of microvascular alterations occur even before eGFR drops below 60 mL/min/1.73 m^2^ [12]. Similarly, in our study which included only participants with eGFR > 60 mL/min/1.73 m^2^, the univariate analysis showed a significant, linear relationship between PI and eGFR. This association was no longer significant after adjustment for other clinical variables. This might be due to our limited sample size or to the limited range of eGFRs.

We found that the phase of the menstrual cycle and the estrogen- or progesterone-levels were not associated with the PI. This is in contrast with animal studies that reported that renal blood flow and RAAS are influenced by estrogens [9,21,22]. Estrogens stimulate NO release by glomerular cells and can thus alter the renal hemodynamics [4,23]. Some animal studies also suggest that sex differences in renal function are linked to differences in the expression of angiotensin type 2 (AT2) receptor, with female rats exhibiting greater renal AT2 protein and mRNA expression compared to male rats [24,25]. Indeed, pharmacological blockade of the AT2 receptor blunts the autoregulation of renal blood flow only in female rats, suggesting that females rely more on the AT2 receptor to control the renal hemodynamics [25]. However, we did not find any correlation between PI, menstrual cycle and sex hormones. In line with this finding, a study examining renal Doppler flow parameters in females between the follicular and the luteal phases of the menstrual cycle did not find differences in RRI, suggesting no impact of sex-related hormonal alterations on renal vascular properties [26]. However, RRI is also influenced by other systemic factors such as HR, BP and arterial stiffness [27].

Interestingly, we found that the PI was significantly and positively associated with PRA, suggesting that the more RAAS is activated, the higher is the renal cortical micro-perfusion. Previous studies in healthy volunteers showed that a pharmacological challenge with angiotensin II (ATII) reduced significantly the PI and RPF in healthy volunteers [11]. However, the amount of infused ATII was much higher than that occurring in a daily setting under physiological conditions. In addition, a potential role of renin in regulating the microcirculation of the kidney cortex, directing the renal blood flow towards the cortex or the inner layers of the cortex, has been hypothesized [28,29]. Furthermore, animal studies showed that the RAAS effect on the renal micro-perfusion is not uniform, with the infusion of ATII producing more vasoconstriction in the inner medulla than in the cortex [4]. Intra-renal regulation of RAAS activation and angiotensin concentrations at the level of the peri-nephronic interstitial fluid and in the tubular fluid has also been described [30,31]. It is therefore possible that the dose–response curve between PRA and renal micro-perfusion is non-linear, with positive effects at low-grade activation and negative ones at intense activation, but this clearly needs more research. Our findings illustrate that the renal micro-circulation is not necessarily controlled by the same factors as the renal macro-circulation, nor in the same way.

Limitations of our study include the unexpected large inter-individual differences in PI (SD > 50% than initially estimated), which makes our study underpowered to fully assess the determinants influencing the PI. Whether this can be explained by differences in systemic and intra-renal RAAS activity, unknown other clinical variables such as the degree of expression of AT1 and AT2 receptors, the integrity of the endothelium or renal sympathetic system activation is still an open question and should be accounted for in future studies [14,32,33,34].

Another limitation is that any interpretation of the relationship between systemic hemodynamics and micro-perfusion may be limited by the fact that different devices for BP measurement were used in the GenderBOLD and BRAIrdN studies. In addition, for technical and economic reasons, we only measured the PI of the right kidney in almost all subjects; it is therefore possible that the results would be different for the left kidneys.

In addition, the PI values were obtained by placing ROIs around the cortex. ROIs are selected by the image analyzer, making them observer-dependent. Moreover, in a previous study, the inter-observer variability was found to be extremely low [12]. Furthermore, in our study there was a similar distribution of the PI among males and females, as well as in volunteers from the two different cohorts, supporting the reproducibility of the method.

## 5. Conclusions

In conclusion, our study showed that sex differences exist in the cortical microcirculation as assessed by CEUS, but these differences are partly explained by differences in eGFR between the sexes. As in a previous study, renal micro-perfusion was associated with eGFR. A role of the menstrual cycle phases and the estradiol or progesterone levels in the regulation of renal hemodynamics was not confirmed, but the associations might be underpowered due to the limited sampling of the hormones. Finally, our results suggest the involvement of PRA in renal cortical perfusion in physiological states. Further studies on the physiology of the renal microcirculation may allow us to understand and early recognize the different trajectories in the progression towards CKD. This study also showed that CEUS is an interesting tool to assess the effect of interventions that potentially reverse the microvascular damage.

## Figures and Tables

**Table 1:** Baseline characteristics of the total population and by sex.

Parameter	Total *n* = 115	Males *n* = 38	Females *n* = 77	*p*
Age (years)	37 ± 12	37 ± 13	37 ± 12	0.81
BMI (kg/m^2^)	24.1 ± 3.4	24.6 ± 3.4	23.8 ± 3.3	0.26
SBP (mmHg)	117 ± 11	124 ± 7	113 ± 10	0.00
DBP (mmHg)	71 ± 8	72 ± 9	70 ± 7	0.34
MBP (mmHg)	86 ± 8	89 ± 7	84 ± 7	0.00
HR (bpm)	66 ± 10	61 ± 10	68 ± 9	0.00
Creatinine (µmol/L)	72 ± 11	82 ± 10	67 ± 9	0.00
eGFR (mL/min/1.73 m^2^)	101 ± 17	91 ± 17	106 ± 15	0.00
Sodium (mmol/L)	140 ± 2	141 ± 1	140 ± 2	0.11
Potassium (mmol/L)	3.81 ± 0.27	3.89 ± 0.25	3.77 ± 0.27	0.03
Uric acid (mmol/L)	279 ± 119	321 ± 56	259 ± 135	0.01
Bicarbonate (mmol/L)	23 ± 2	24 ± 2	23 ± 1	0.00
PRA (ng/mL/h)	0.77 (0.33–1.19)	0.40 (0.15–0.87)	0.93(0.54–1.27)	0.001
PAC (pmol/L)	127 (69–222)	81 (34–149)	164 (94–278)	0.0002

Data are presented as mean ± SD or median (IQR) depending on the normality of the distribution; BMI: body mass index; SBP: systolic blood pressure; DBP: diastolic blood pressure; MBP: mean blood pressure; HR: heart rate; eGFR: estimated by CKD-EPI formula glomerular filtration rate; PRA: plasma renin activity; PAC: plasma aldosterone concentration.

**Table 2:** Renal ultrasound parameters of the study population by sex.

Parameters	Males *n* = 38	Females *n* = 77	*p*
PI (a.u)	1965 (1294–3346)	2705 (1641–3777)	0.02
rBV (a.u.)	4149 (2386–6562)	4895 (3330–7020)	0.21
mTT (s)	2.15 (1.7–2.7)	1.85 (1.53–2.34)	0.09
RRI (a.u.)	0.60 (0.56–0.64)	0.63 (0.60–0.66)	0.0003
Kidney Volume (mL)	129 (97–159)	109 (94–129)	0.04

Data are presented as median (IQR). PI: perfusion index; rBV: renal blood volume; mTT: mean transit time; RRI: renal resistive index.

**Table 3:** Renal ultrasound parameters according to the menstrual phase.

Parameter	Follicular *n* = 15	Luteal *n* = 11	*p*
PI (a.u.)	2857 (1445–4748)	2571 (2224–3604)	0.83
rBV	4895 (2872–7010)	4982 (3322–5800)	0.92
mTT (s)	1.68 (1.53–2.09)	1.81 (1.50–2.34)	0.84
RRI	0.61 (0.60–0.66)	0.64 (0.62–0.65)	0.31

Data are presented as median (IQR). PI: perfusion index; rBV: renal blood volume; mTT: mean transit time; RRI: renal resistive index.

**Table 4:** Multivariate regression analysis including all the variables that were significantly associated with the outcome variable PI (Log) in univariate analysis, showing the associations between the perfusion index (PI) (outcome variable) and clinical variables.

Clinical Variables	Fully Adjusted β (95% CI)	*p*
Sex (Female vs. Male)	0.065 (−0.33 to 0.46)	0.74
eGFR (mL/min/1.73 m^2^)	0.002 (−0.008 to 0.011)	0.69
SBP (mmHg)	−0.007 (−0.02 to 0.10)	0.43
HR (bpm)	0.005 (−0.011 to 0.02)	0.55
Potassium (mmol/L)	−0.22 (−0.74 to 0.30)	0.40
Bicarbonates (mmol/L)	−0.05 (−0.15 to 0.05)	0.35
PRA-Log (ng/mL/h)	0.17 (0.01 to 0.33)	0.04

CI: confidence interval; SBP: systolic blood pressure; eGFR: estimated by CKD-EPI formula glomerular filtration rate; HR: heart rate; PRA: plasma renin activity.

## Data Availability

All data generated or analyzed during this study are included in this article and its online Supplementary Material. Further inquiries can be directed to the corresponding author.

## Supplementary Materials

The following supporting information can be downloaded at: https://www.mdpi.com/article/10.3390/jcm12124141/s1, Figure S1: Distribution of perfusion between GenderBOLD and BRAIrdDN study, Figure S2: Logarithmic transformation of distribution of PI (Kernel density) by sex; Table S1: Differences between GenderBOLD and BRAIrdN study, Table S2: Correlation of the Perfusion Index, Renal Blood volume and Mean Transit Time with the variables of interest, Table S3: HS GenderBOLD and BRAIrdN-Multivariate regression analysis including all the variables that were significantly associated with the outcome variable PI (Log) in univariate analysis, showing the associations between the perfusion index (PI) (outcome variable) and clinical variables.
