# Supplementary material for: Determinants of Renal Micro-Perfusion as Assessed with Contrast-Enhanced Ultrasound in Healthy Males and Females
Source: J Clin Med. 2023 Jun 20;12(12):4141. doi: 10.3390/jcm12124141 (PMC10298862; doi:10.3390/jcm12124141)
Supplement: Supplementary file 1 [file jcm-12-04141-s001.zip › jcm-2360789-SI.pdf]

**Supplementary material**

- Suppl. Figure S1- Distribution of perfusion between GenderBOLD and BRAIrdN study
- Suppl. Figure S2 – Logarithmic transformation of distribution of PI
- Suppl. Table S1- Differences between GenderBOLD and BRAIrdN study
- Suppl. Table S2- Association of the Perfusion Index with the variables of interest
- Suppl. Table S3- HS GenderBOLD and BRAIrdN - Multivariate regression analysis including all the variables that were significantly associated with the outcome variable PI (Log) in univariate analysis, showing the associations between the perfusion index (PI) (outcome variable) and clinical variables

**Supplementary Figure S1 -** Distribution of perfusion between GenderBOLD and BRAIrdDN study

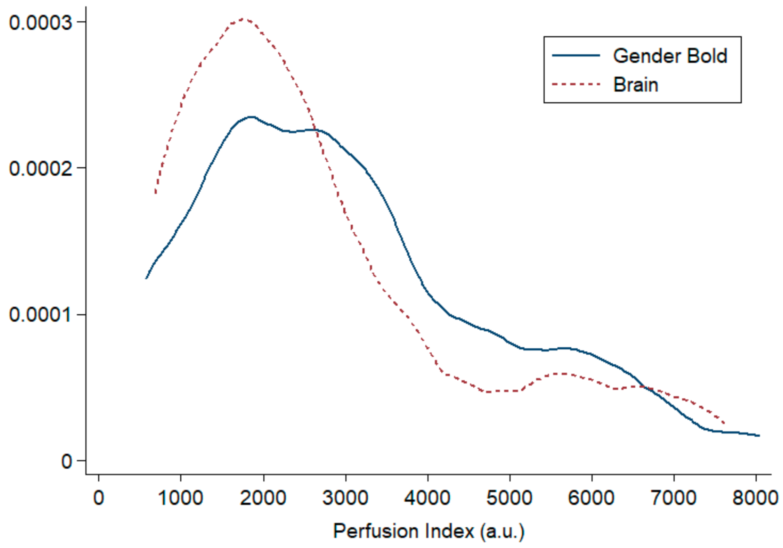

**Supplementary Figure S2 –** Logarithmic transformation of distribution of PI (Kernel density) by sex

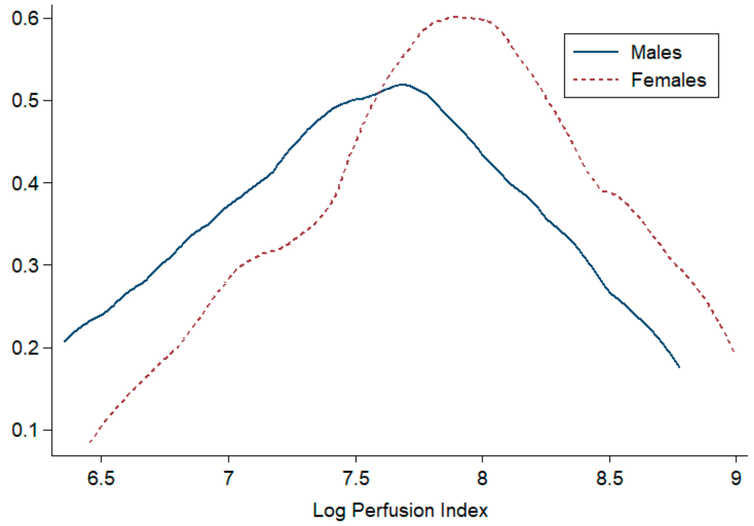


**Supplementary Table S1**- Differences between GenderBOLD and BRAIrdN study

| Clinical Variables | GenderBOLD | BRAIrdN | p |
|---|---|---|---|
| Age (years) | 39±13 | 34±11 | 0.07 |
| BMI (kg/m²) | 24±4 | 24±3 | 0.20 |
| SBP (mmHg) | 114±11 | 120±8 | **0.00** |
| DBP (mmHg) | 71±8 | 71±7 | 0.92 |
| MBP (mmHg) | 85±8 | 87±6 | 0.18 |
| HR (bpm) | 68±8 | 62±11 | **0.00** |
| Creatinine (µmol/l) | 69±10 | 76±12 | **0.00** |
| eGFR (ml/min/1.73m²) | 102±15 | 99±21 | 0.41 |
| Sodium (mmol/l) | 140±1 | 141±1 | **0.00** |
| Potassium (mmol/l) | 3.81±0.2 | 3.81±0.3 | 0.89 |
| Uric acid (mmol/l) | 277±142 | 283±64 | 0.81 |
| Bicarbonate (mmol/l) | 23±1 | 23±2 | 0.45 |
| PI (a.u.) | 2600(1539-3777) | 2011(1357- 3291) | 0.24 |
| rBV | 4990(3322- 6624) | 4001(2545- 7020) | 0.36 |
| mTT (seconds) | 1.96(1.57-2.35) | 1.88(1.53- 2.67) | 0.89 |
| RRI | 0.63(0.60-0.66) | 0.59(0.56-0.64) | **0.006** |
| Kidney volume (ml) | 125(99-145) | 101(89-125) | **0.005** |
| PRA (ng/ml/h) | 1.05(0.68-1.58) | 0.38(0.17-0.77) | **0.000** |
| PAC (pmol/l) | 181(95-275) | 94(56-137) | **0.005** |

Data are expressed as mean±SD or median(IQR). BMI: body mass index; SBP : systolic blood pressure; DBP : diastolic blood pressure; MBP : mean blood pressure; HR: heart rate; eGFR: estimated by CKD-EPI formula glomerular filtration rate PI: perfusion index; rBV: renal blood volume; mTT: mean transit time; RRI: renal resistive index; PRA: plasma renin activity; PAC: plasma aldosterone concentration

**Supplementary Table S2**- Correlation of the Perfusion Index, Renal Blood volume and Mean Transit Time with the variables of interest

| Clinical Variables | PI | | rBV | | mTT | |
|---|---|---|---|---|---|---|
| | **Rho** | **p** | **Rho** | **p** | **Rho** | **p** |
| Female sex | 0.20 | **0.03** | 0.08 | 0.40 | -0.12 | 0.21 |
| Age (years) | -0.02 | 0.86 | 0.03 | 0.75 | 0.12 | 0.20 |
| BMI (kg/m²) | -0.02 | 0.80 | 0.00 | 0.96 | 0.08 | 0.39 |
| SBP (mmHg) | -0.19 | **0.04** | -0.06 | 0.54 | 0.24 | **0.01** |
| DBP (mmHg) | 0.02 | 0.86 | 0.09 | 0.32 | 0.19 | **0.04** |
| MBP (mmHg) | -0.07 | 0.45 | 0.05 | 0.63 | 0.25 | **0.01** |
| HR (bpm) | 0.25 | **0.01** | 0.12 | 0.20 | -0.18 | 0.05 |
| Creatinine (µmol/l) | -0.17 | 0.07 | -0.11 | 0.24 | 0.18 | 0.05 |
| eGFR (ml/min/1.73m²) | 0.20 | **0.03** | 0.15 | 0.12 | -0.15 | 0.11 |
| Sodium (mmol/l) | 0.02 | 0.82 | 0.08 | 0.38 | 0.12 | 0.22 |
| Potassium (mmol/l) | -0.24 | **0.01** | -0.18 | 0.05 | 0.13 | 0.17 |
| Uric acid (mmol/l) | -0.01 | 0.94 | 0.01 | 0.90 | 0.04 | 0.67 |
| Bicarbonate (mmol/l) | -0.20 | **0.03** | -0.16 | 0.08 | 0.03 | 0.76 |
| RRI | 0.04 | 0.70 | 0.00 | 0.97 | -0.04 | 0.71 |
| Kidney volume (ml) | -0.01 | 0.95 | -0.07 | 0.49 | -0.08 | 0.43 |
| PRA (ng/ml/h) | 0.37 | **0.001** | 0.37 | **0.00** | -0.03 | 0.80 |
| PAC (pmol/l) | 0.22 | **0.04** | 0.20 | 0.06 | -0.04 | 0.68 |

BMI: body mass index; SBP : systolic blood pressure; DBP : diastolic blood pressure; MBP : mean blood pressure; HR: heart rate; eGFR: estimated by CKD-EPI formula glomerular filtration rate PI: perfusion index; rBV: renal blood volume; mTT: mean transit time; RRI: renal resistive index; PRA: plasma renin activity; PAC: plasma aldosterone concentration

**Supplementary Table S3 -** HS GenderBOLD and BRAIrdN - Multivariate regression analysis including all the variables that were significantly associated with the outcome variable PI (Log) in univariate analysis, showing the associations between the perfusion index (PI) (outcome variable) and clinical variables

| Clinical Variables | Univariate analysis β (95% CI) | p | Multivariate analysis β (95% CI) | p |
|---|---|---|---|---|
| eGFR (ml/min/1.73m²) | 0.13(0.004 to 0.21) | 0.004 | 0.01(-0.0007 to 0.02) | 0.068 |
| Bicarbonates (mmol/l) | -0.10(-0.19 to -0.01) | 0.03 | - 0.07(-0.19 to 0.05) | 0.24 |
| PRA - Log (ng/ml/h) | 0.21(0.034 to 0 .38) | 0.02 | 0.17(-0.05 to 0.31) | 0.14 |

CI: confidence interval; eGFR: estimated by CKD-EPI formula glomerular filtration rate; PRA: plasma renin activity
